# Similarity and Diversity of Presynaptic Molecules at Neuromuscular Junctions and Central Synapses

**DOI:** 10.3390/biom12020179

**Published:** 2022-01-21

**Authors:** Kenji Takikawa, Hiroshi Nishimune

**Affiliations:** 1 Laboratory of Neurobiology of Aging, Tokyo Metropolitan Institute of Gerontology, 35-2 Sakaecho, Itabashi-ku, Tokyo 173-0015, Japan; takikawa@tmig.or.jp; 2 Department of Applied Biological Science, Tokyo University of Agriculture and Technology, 3-8-1 Harumi-cho, Fuchu-shi, Tokyo 183-8538, Japan

**Keywords:** active zone, aged, Bassoon, GABA, glutamate, Munc13, neuromuscular junction, release probability, super-resolution microscopy

## Abstract

Synaptic transmission is essential for controlling motor functions and maintaining brain functions such as walking, breathing, cognition, learning, and memory. Neurotransmitter release is regulated by presynaptic molecules assembled in active zones of presynaptic terminals. The size of presynaptic terminals varies, but the size of a single active zone and the types of presynaptic molecules are highly conserved among neuromuscular junctions (NMJs) and central synapses. Three parameters play an important role in the determination of neurotransmitter release properties at NMJs and central excitatory/inhibitory synapses: the number of presynaptic molecular clusters, the protein families of the presynaptic molecules, and the distance between presynaptic molecules and voltage-gated calcium channels. In addition, dysfunction of presynaptic molecules causes clinical symptoms such as motor and cognitive decline in patients with various neurological disorders and during aging. This review focuses on the molecular mechanisms responsible for the functional similarities and differences between excitatory and inhibitory synapses in the peripheral and central nervous systems, and summarizes recent findings regarding presynaptic molecules assembled in the active zone. Furthermore, we discuss the relationship between functional alterations of presynaptic molecules and dysfunction of NMJs or central synapses in diseases and during aging.

## 1. Introduction

At chemical synapses in the neuromuscular junctions (NMJs) and central nervous systems, synaptic transmission is achieved by neurotransmitters released from presynaptic terminals and received by postsynaptic cells. Neurotransmitter release occurs through the docking, priming, and fusion of synaptic vesicles in the active zones of the presynaptic terminal, a thickened membrane region containing cytosolic electron-dense materials [1,2]. Synaptic vesicles are stored in the reserve pool of the presynaptic terminal, docked on the presynaptic membrane near the active zone using the soluble N-ethylmaleimide-sensitive factor attachment protein receptor (SNARE) complex that includes synaptobrevin on the synaptic vesicle membrane and syntaxin and synaptosome-associated protein-25 (SNAP-25), and primed to fuse immediately with presynaptic membrane in response to an increase in the intracellular Ca^2+^ concentration. Numerous molecules have been identified to accumulate in the active zone and play various roles in neurotransmitter release. Furthermore, the use of super-resolution microscopy to obtain spatial information about presynaptic molecules on the nanometer scale [3,4,5,6,7] and fluorescence probes to visualize neurotransmitter release at individual synapses [6,7,8] have revealed the molecular mechanisms underlying the regulation of neurotransmitter release by presynaptic molecules. Dysfunction of the presynaptic molecules causes clinical symptoms, such as motor and cognitive decline in individuals with neurological diseases and during aging [9,10,11,12,13]. This review is composed of two sections discussing recent findings on presynaptic molecules assembled in the active zone. Section 1 highlights the similarities and differences in presynaptic structures and molecules at NMJs and central synapses. Section 2 discusses the dysfunction of presynaptic molecules in neurological diseases and during aging.

## 2. Similarities and Differences in Presynaptic Structure and Molecules at NMJs and Central Synapses

Couteaux and Pécot-Dechavassine identified a thickened membrane region containing cytosolic electron-dense materials where synaptic vesicles fuse with the membrane using electron microscopy, and named it the “active zone” [1]. Synaptic vesicles containing neurotransmitters dock, fuse, and release neurotransmitters into the synaptic cleft due to an increase in the intracellular Ca^2+^ concentration at the active zones, which make them an essential structure for the spatial control of synaptic transmission [2]. Interestingly, although the size of presynaptic terminals differs among NMJs and central synapses [14], the size of individual active zones and the types of presynaptic molecules that accumulate in the active zone are conserved among NMJs and central synapses. Furthermore, differences in the nanometer-scale spatial arrangements of presynaptic molecules are related to the diversity of functions of NMJs and central synapses. In this section, we will compare NMJs and central synapses with respect to the size and number of active zones and the type, function, and spatial arrangement of presynaptic molecules that accumulate in the active zones, and provide an overview of how these parameters relate to similarities and differences in presynaptic functions.

### 2.1. Number and Size of Active Zones at NMJs and Central Nervous System Synapses

The number of active zones per presynaptic terminal is important, as it is closely related to the efficacy of synaptic transmission [15,16] and is one of the factors that contributes to the diversity of synaptic transmission functions among different types of synapses [14]. The number of active zones per presynaptic terminal at NMJs is approximately 780 to 850 in adult mice [15,17]. Furthermore, the synaptic terminal area of NMJs in these mice is 295 to 335.9 μm^2^ [15,17], indicating that the density of active zones is 2.5–2.6/μm^2^. In NMJs of adult humans, the nerve terminal area was determined to be 122.7 μm^2^ in a study using immunohistochemistry [18], and the density of active zones was identified as 2.6/μm^2^ in another study using electron microscopy [19]. Interestingly, the active zone density at mammalian NMJs seems to be maintained at approximately 2.3–2.7 active zones/μm^2^ [14]. Additionally, a positive correlation was observed between the area of synaptic terminals at NMJs and quantal content (the number of synaptic vesicles released per action potential) between vertebrates with synaptic terminals of different sizes (e.g., 335.9 μm^2^ in mice and 122.7 μm^2^ in humans) [16]. Based on these results, the density of active zones is essential for determining the efficacy of synaptic transmission.

One active zone per presynaptic terminal is present at more than 90% of synapses in the stratum radiatum of the hippocampal CA1 region and piriform cortex layers 1a and 1b [20,21]. These central synapses have small bouton volumes: 0.086 μm^3^ in the hippocampus, 0.367 μm^3^ in piriform cortex layer 1a, and 0.208 μm^3^ in piriform cortex layer 1b [20,21]. On the other hand, the presynaptic terminal area of the calyx of Held, an axon terminal extending from the cochlear nucleus to the medial nucleus of the trapezoid body in the brainstem, is approximately 1022 μm^2^ in postnatal day nine rats, which is greater than that of NMJs [22]. The number of active zones per presynaptic terminal in the calyx of Held is approximately 554 [22], which is an exceptionally large number among central synapses [14]. However, the area of a single active zone is similar between NMJs and central synapses: 0.147 μm^2^ for rat diaphragm NMJs [23], 0.082 μm^2^ for mouse sternocleidomastoid NMJs [17], 0.039 μm^2^ in the hippocampus [20], 0.095 μm^2^ and 0.097 μm^2^ in layer 1a and layer 1b of the piriform cortex, respectively, and 0.089 μm^2^ in the calyx of Held [21,24]. In addition, the active zone area rarely exceeds 0.4 μm^2^ in neck motoneurons, and large synapses form multiple active zones without exceeding this size limit [25]. Thus, variations in the number of active zones may lead to the diversification of synaptic transmission functions at NMJs and central synapses (Table 1).

### 2.2. Synaptic Proteins in Active Zones

**Figure 1:**
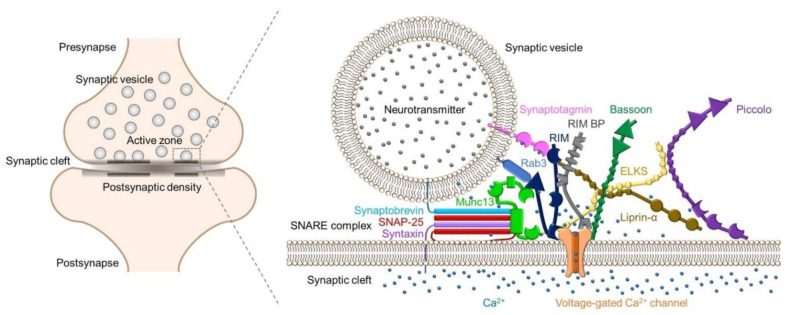
Schematic representation of the presynaptic protein complex containing SNARE proteins, active zone proteins, synaptic vesicle proteins, and voltage-gated calcium channels in an active zone.

Studies of central synapses have identified active zone proteins such as Bassoon [26], CAST/ELKS [27,28], Munc13s [29], Piccolo [30], and RIMs [31]. These active zone proteins, as well as SNARE proteins, voltage-gated calcium channels (VGCCs), and Ca^2+^ sensor proteins, are conserved at NMJs, indicating that the components of presynaptic terminals are similar between central synapses and NMJs [32,33,34,35,36] (Figure 1). Knockout mice and knockdown cells revealed the roles of these active zone proteins in synaptic functions (Table 2).

In *Rim1* and *Rim2* conditional double knockout mice, the number of docked synaptic vesicles per active zone was reduced by approximately 70% at the calyx of Held synapses, according to an electron microscopy study [39]. In addition, the size of the readily releasable pool (RRP) was reduced by 75% [39]. These results are consistent with the observation that RIMs bind to the synaptic vesicle protein Rab3s [31], the active zone protein Munc13s [40,48,49,50], Liprin-α [51], and RIM-binding proteins [52,53]. In addition, the presynaptic Ca^2+^ current decreases by 50% [39]. The decrease in Ca^2+^ currents due to null mutations in *Rim* genes reflects the fact that RIMs bind directly to the α1-subunit of P/Q-type and N-type VGCCs and β4-subunit of VGCCs, tether VGCCs to active zones, and maintain Ca^2+^ influx through ion channels [41,54]. The decrease in RRP and Ca^2+^ currents resulted in an approximately 25% decrease in the release probability of synaptic vesicles and a 1/5-fold decrease in the excitatory postsynaptic current (EPSC) amplitude [39]. However, the amplitude of miniature EPSCs was unchanged [39]. This result suggests that RIMs do not affect the formation of individual synaptic vesicles.

In cultured hippocampal neurons from *Rim1* and *Rim2* conditional double knockout mice, the number of docked synaptic vesicles per active zone was reduced by approximately 1/2-fold, and the RRP size was reduced more than 1/3-fold compared to those from control mice, similar to the findings from the calyx of Held [41]. In contrast, neurons from double knockout mice exhibited an overall normal morphology with unchanged synapse size and density [41]. The amplitudes of miniature EPSCs and inhibitory postsynaptic currents (IPSCs) were similar, but the frequencies of miniature EPSCs and IPSCs were reduced 1/10- and 1/3-fold, respectively [40]. In addition, the amplitudes of evoked EPSCs and IPSCs were approximately 1/10-fold smaller [41]. A reduced release probability was observed at inhibitory synapses in cultured hippocampal neurons of *Rim1* and *Rim2* conditional double knockout mice and at excitatory and inhibitory synapses in acute hippocampal CA1 slices from *Rim1a* and *Rim1b* conditional knockout mice [37,40].

At NMJs in *Rim1a* and *Rim2a* double knockout embryos, the miniature endplate potential (EPP) amplitude was not significantly different from control embryos from the same litter as in central synapses [33]. The evoked EPP amplitude was approximately 1/10-fold smaller in double knockout mice than in control mice [33]. Furthermore, the failure rate increased dramatically in the double knockout group, which showed 115 failures per 518 stimuli (22.2%) compared to the control group, which showed only one failure per 309 stimuli (0.32%) [33]. Thus, the effects of RIMs on the evoked release amplitude and the release probability are very similar between NMJs and central synapses. These similar effects of RIMs on synaptic release suggest that RIMs contribute to the priming of synaptic vesicles and to the maintenance of VGCC function in achieving high Ca^2+^ concentrations in the vicinity of synaptic vesicles at NMJs and central synapses.

The Munc13 protein family consists of four highly homologous members, including Munc13-1/2/3/4 [2,55]. In mammals, two splice variants of Munc13-2 have been identified: bMunc13-2 that is expressed specifically in the brain, and ubMunc13-2 that is expressed ubiquitously in the body, including the brain and NMJs [29,32,48,56]. The Munc13 protein family is essential for exocytosis, but the effects of Munc13 proteins on synaptic release function and synaptic ultrastructure partially differ between central synapses and NMJs.

Munc13-1 and ubMunc13-2 are recruited to presynaptic active zones via the binding of RIM to their highly homologous N-terminal domains [48,50]. On the other hand, bMunc13-2 is recruited to presynaptic active zones via the interaction of the unique coiled-coil domains of its N-terminus with ELKS1 [57]. Furthermore, the RIM binding region of Munc13-1 and the ELKS1 binding region of bMunc13-2 are required for efficient synaptic vesicle priming [48,57]. Thus, members of the Munc13 protein family are recruited to active zones through specific interactions with RIM and ELKS1 to achieve efficient synaptic vesicle priming.

Munc13-1 and Munc13-2 have been suggested to underlie the diversity of neurotransmitter release properties at excitatory and inhibitory synapses [43,58]. Excitatory glutamatergic synapses formed by cultured hippocampal neurons derived from *Munc13-1* knockout mice showed markedly reduced neurotransmitter release in response to stimuli such as evoked action potentials, exposure to calcium ionophores, and hypertonic sucrose solutions [42]. Therefore, Munc13-1 is essential for vesicle release at most excitatory glutamatergic synapses. However, a small subpopulation of glutamatergic synapses was not affected by the loss of Munc13-1, and vesicle release at glutamatergic synapses was completely abolished only in *Munc13-1* and *Munc13-2* double knockout mice [43], which had a normal number of synapses, but fewer synaptic vesicles docked in active zones [59,60]. Thus, these small subpopulations probably depend on Munc13-2 for their vesicle release function or may express both Munc13-1 and 13-2 [57] and depend on both Munc13 proteins. Furthermore, Munc13-1- or ub/bMunc13-2-expressing synapses showed different types of short-term synaptic plasticity during high-frequency stimulation [57,58]. Rescue of *Munc13-1* expression in cultured hippocampal neurons derived from *Munc13-1* and *Munc13-2* double knockout mice decreased EPSC amplitudes during 10 Hz stimulation [58]. Conversely, rescue of *ubMunc13-2* or *bMunc13-2* expression in cultured hippocampal neurons derived from *Munc13-1* and *Munc13-2* double knockout mice increased EPSC amplitudes during 10 Hz stimulation [57,58]. In summary, Munc13-1 and Munc13-2 might promote short-term depression and facilitation, respectively, at excitatory synapses during 10 Hz stimulation.

In contrast, inhibitory GABAergic synaptic transmission was not altered by the absence of either Munc13-1 or Munc13-2 alone, but was completely abolished in cultured hippocampal neurons derived from *Munc13-1* and *Munc13-2* double knockout mice [43]. Thus, Munc13-1 and Munc13-2 have redundant functions at GABAergic synapses. Furthermore, IPSC amplitudes in cultured hippocampal neurons derived from *Munc13-1* knockout mice were reduced to similar levels as those in wild-type neurons during 10 Hz stimulation [58]. This result may also reflect the redundancy of Munc13-1 and Munc13-2 at GABAergic synapses [43].

**Figure 2:**
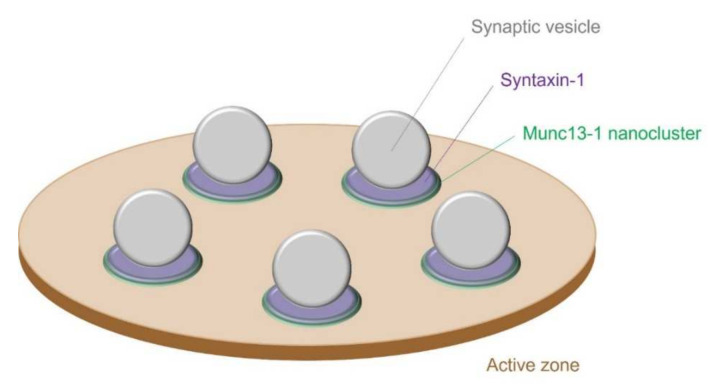
Model of Munc13-1 nanoclusters forming synaptic vesicle release sites. The Munc13-1 nanocluster (green) recruits syntaxin 1 (magenta) and forms a synaptic vesicle release site. The number of synaptic vesicle release sites corresponds almost exactly to the number of Munc13-1 nanoclusters, showing a one-to-one relationship.

Interestingly, the number of Munc13-1 nanoclusters matches the number of release sites in cultured rat hippocampal glutamatergic synapses [7]. Furthermore, Munc13-1 clusters are formed by supramolecular self-assembly, and the SNARE protein syntaxin 1, which is essential for synaptic vesicle exocytosis, is recruited to each Munc13-1 cluster [7] (Figure 2). Intriguingly, the number of functionally analyzed release sites for each synapse ranged from 1 to 18, with an average of 4.9 [7]. Most CNS synapses have a single active zone detected by electron microscopy; however, multiple Munc13-1 clusters may serve as a mechanism for the release of multiple synaptic vesicles in response to a single action potential.

In GABAergic synapses formed by molecular layer interneurons in cerebellar slices, the number of functional release sites ranges from one to six per synapse [61]. Researchers have not yet clearly determined which molecule determines the number of release sites at GABAergic synapses. Since the functions of Munc13-1 and Munc13-2 are completely redundant in hippocampal GABAergic synapses [43], and Munc13-3 is also expressed in the cerebellum [62], a fascinating approach would be to study whether the number of release sites is defined by the number of Munc13 family protein clusters at GABAergic synapses.

At NMJs of *Munc13-1* and *Munc13-2* double knockout mice, the miniature EPP frequency is significantly increased (more than two-fold), but the miniature EPP amplitude is normal [32]. Furthermore, the failure rate increased dramatically to 77.1% in the double knockout mice compared to the controls at 0.7%. As a result, the EPP amplitude is significantly reduced by approximately 1/16-fold compared to controls [32]. In addition, docked synaptic vesicles were occasionally observed at NMJ active zones in these double knockout mice using electron microscopy [32]. These results suggest the importance of the Munc13 protein family in synaptic vesicle priming at NMJs. Interestingly, the residual synaptic transmission suggests that priming at NMJs is partly Munc13 independent, which is different from most central synapses. In summary, Munc13 is essential for exocytosis at NMJs and central synapses.

Two genes of the ELKS protein family, *ELKS1 (CAST2)* and *ELKS2 (CAST1)*, have been identified [27,28]. *ELKS1* is expressed at central synapses [63] and NMJs [64] as well as in various tissues and cells, such as pancreatic β-cells [65], while *ELKS2* is mainly expressed at central synapses [64]. ELKS1 and ELKS2 differentially affect neurotransmitter release properties at excitatory and inhibitory synapses in the hippocampus.

In cultured hippocampal neurons and acute hippocampal slices from *ELKS2a* constitutive and conditional knockout mice, neurotransmitter release properties were unaffected or only mildly affected at excitatory synapses [44]. In contrast, the evoked IPSC amplitude was increased at inhibitory synapses [44]. In addition, this effect was accompanied by an increase in RRP size and the release probability of synaptic vesicles [44]. Therefore, ELKS2a seems to act specifically on inhibitory neurons, limiting the RRP size and release probability of synaptic vesicles. Another possibility is that loss of ELKS2a promotes the priming function of ELKS1 and bMunc13-2, as described in the seven previous paragraphs, resulting in increased RRP size [57] because the expression level of ELKS1 increased approximately five-fold in the synaptic membrane fractions of ELKS2 knockout mouse brains [66].

In cultured hippocampal neurons derived from *ELKS1a* and *ELKS2a* conditional double knockout mice, the frequency of spontaneous release at excitatory and inhibitory synapses and the amplitudes of evoked EPSCs and IPSCs were reduced by approximately 50% [46,47]. Surprisingly, the mechanisms responsible for these decreases in postsynaptic current amplitudes differed. The loss of ELKS1a and ELKS2a decreases the RRP size, but does not change presynaptic Ca^2+^ influx or the release probability of synaptic vesicles at excitatory synapses [46]. In contrast, at inhibitory synapses, the loss of ELKS1a and ELKS2a reduces presynaptic Ca^2+^ influx and the release probability of synaptic vesicles, but does not change the RRP size [46,47]. Based on these results, ELKS increases the RRP size at excitatory synapses rather than Ca^2+^ influx and release probability at inhibitory synapses. Furthermore, rescue experiments revealed that the RRP size at excitatory synapses is regulated by the N-terminal Liprin-α- and Bassoon-binding domains of ELKS, but not by the C-terminal RIM-binding domain [46].

In slices of the calyx of Held, no change in EPSC amplitude was observed between the control and *ELKS1* conditional and *ELKS2* null double knockout mice [45], but the frequency of spontaneous release was increased in the slices from double knockout mice [45]. Furthermore, the RRP size was reduced, but the release probability of synaptic vesicles was increased [45]. Therefore, the effects of ELKS1 and ELKS2 on neurotransmitter release are very different between the hippocampal synapses and the calyx of Held synapses.

*ELKS1* is expressed at NMJs, but *ELKS2* has not been detected, unlike central synapses [64]. Although the physiological role of ELKS in NMJs remains unknown, the results from studies of *Drosophila* NMJs may provide insights into the function of ELKS in mammalian NMJs. The mutation of the *Drosophila* ELKS homolog *bruchpilot (brp)* leads to complete loss of the T-bar structure of the *Drosophila* active zone and substantially reduces the clustering of Ca^2+^ channels, resulting in reduced evoked excitatory junctional current amplitudes [67]. The function of ELKS1 in pancreatic β-cells may provide another hint to understand the function of ELKS1 in NMJs. Pancreatic β-cells secrete insulin through exocytosis triggered by Ca^2+^ influx from L-type VGCCs [65]. However, knockout of the *ELKS1* gene in pancreatic β-cells inhibits glucose-stimulated insulin secretion and decreases the current density of L-type VGCCs [65]. A reduction in Ca^2+^ signaling and evoked release amplitude was also observed at the central synapses of *ELKS1* and *ELKS2* double knockout mice [45,46,47]. Furthermore, ELKS1 interacts with β4-subunit of VGCCs [68]. Taken together, ELSK1 may have a functional role in evoked synaptic vesicle release at mammalian NMJs.

### 2.3. VGCC Types and Clusters in Active Zones

Three types of VGCCs accumulate in presynaptic terminals and induce neurotransmitter release by Ca^2+^ influx: P/Q-type, N-type, and R-type. The developmental switching of VGCC types occurs at several central synapses and NMJs, and differences in the types of VGCCs expressed at mature synapses lead to differences in the release properties of synaptic vesicles.

In the calyx of Held synapses of developing rats at postnatal days 7 to 10, P/Q-type, N-type, and R-type VGCCs are utilized, while at postnatal day 13, P/Q-type VGCCs are predominantly utilized [69,70]. On the other hand, P/Q-type and N-type VGCCs or all three types of VGCCs are maintained at several types of mature excitatory synapses in the cerebral cortex and hippocampus, respectively [70,71]. For example, a single mossy fiber bouton of rat hippocampal slices contains an average of 1300 P/Q-type, 500 N-type, and 160 R-type VGCCs [71]. As mossy fiber boutons contain an average of 29.75 active zones [72], the numbers of these VGCCs per active zone correspond to 44 P/Q-type VGCCs, 17 N-type VGCCs, and 5 R-type VGCCs. Furthermore, P/Q-type VGCCs are activated more efficiently than other VGCCs in response to suprathreshold presynaptic voltage signals, and R-type VGCCs are activated in response to subthreshold signals at single mossy fiber boutons [71].

GABAergic synapses in the cerebellum and thalamus of developing rats at postnatal day 7 utilize P/Q-type and N-type VGCCs, whereas P/Q-type VGCCs are predominantly utilized at postnatal days 16–19 [70,73]. GABA release at two types of GABAergic synapses projecting to dentate gyrus granule cells in rat hippocampal slices is triggered by P/Q-type VGCCs at the presynaptic terminals of parvalbumin-positive neurons and N-type VGCCs at the presynaptic terminals of cholecystokinin-positive neurons [74]. Differences in the expression of VGCC types possibly contribute to differences in the properties of synchronous GABA release in parvalbumin-positive GABAergic neurons, and asynchronous GABA release in cholecystokinin-positive GABAergic neurons in response to presynaptic action potentials [74].

NMJs in developing rodents at postnatal days 0 to 4 utilize P/Q-type and N-type VGCCs [75], whereas P/Q-type VGCCs are predominantly utilized in rodents after postnatal days 5 to 11 and in mature humans [75,76]. Thus, developmental switching of VGCC types occurs at NMJs and at central synapses, and P/Q-type VGCCs become the main VGCCs at these synapses.

Immunoelectron microscopy and super-resolution microscopy have shown that P/Q-type VGCCs are not distributed uniformly, but form multiple nanoclusters in the active zone of the calyx of Held synapses, hippocampal synapses, cerebellar synapses, and NMJs [4,5,77,78,79,80,81,82]. The number of these P/Q-type VGCC clusters may be an important parameter to determine the number of synaptic vesicle release sites. One cluster of P/Q-type VGCCs consists of an average of nine P/Q-type VGCCs in the active zone of excitatory synapses between parallel fibers and molecular layer interneurons in the cerebellum [79]. Intriguingly, at single synapses in cerebellar slices from two-week-old mice, the number of functionally defined release sites ranges from 1 to 10, and the number of P/Q-type VGCC clusters ranges from 1 to 8, with a ratio of 0.83 [79]. Thus, the average number of synaptic vesicle release sites seems to match the number of VGCC clusters [79]. Furthermore, this result supports the multivesicular release hypothesis [83], which suggests that multiple vesicles are released simultaneously from a single synapse in response to a single action potential. Likewise, P/Q-type VGCC clusters have been identified at glutamatergic and GABAergic presynaptic terminals in the stratum radiatum of the hippocampal CA1 area [82]. Moreover, a quantitative morphological analysis revealed a high degree of structural similarity between excitatory and inhibitory synapses in terms of the clustering of P/Q-type VGCCs and the average number of these channels within the synapse [82]. Therefore, the number of release sites at excitatory and inhibitory synapses is likely based on the number of P/Q-type VGCC clusters. In NMJs, numerous P/Q-type VGCC clusters exist per synapse [5], and a single P/Q-type VGCC cluster is predicted to localize at a docking site for two synaptic vesicles at active zones, as described in detail below in Section 2.4. Therefore, the number of VGCC clusters may be an important parameter for estimating the number of synaptic vesicle release sites at central synapses and NMJs.

### 2.4. Spatial Arrangement of VGCCs and Active Zone Proteins

Exocytosis is initiated when Ca^2+^ influx and diffusion from VGCCs to presynaptic terminals are sensed by Ca^2+^ sensor proteins [84,85,86,87]. Therefore, the distance between VGCCs and Ca^2+^ sensor proteins is a critical factor regulating the efficiency of exocytosis. Interestingly, studies using a combination of BAPTA (fast Ca^2+^-binding kinetics, association rate constant of 4.5 × 10^8^ M^−1^ s^−1^) and EGTA (slow Ca^2+^-binding kinetics, association rate constant of 2.7 × 10^6^ M^−1^ s^−1^) [88] have estimated that Ca^2+^ sensor proteins are located at different distances from VGCCs at central synapses and NMJs.

**Figure 3:**
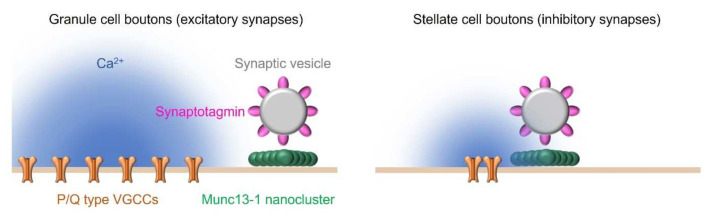
Differences in the distances between synaptic proteins allow synapse-specific regulation of the release probability at cerebellar synapses. Munc13-1 was located approximately 50 nanometers from P/Q-type VGCCs in granule cell boutons. In contrast, Munc13-1 was tightly coupled to a cluster of P/Q-type VGCCs in stellate cell boutons (approximately 10 nanometers). The distance between Munc13-1 and P/Q-type VGCCs at these synapses results in a lower probability of synaptic vesicle release at granule cell synapses and a higher release probability at stellate cell synapses. This figure is adopted with modifications from Liu et al., Neuron 2019, 104, 627 [98] (with permission from Elsevier, Inc., license number: 5191011347259).

In parvalbumin-positive neurons from the rat hippocampal dentate gyrus, BAPTA attenuates synaptic GABA release, but EGTA has no effect on synaptic GABA release [80]. These results indicate that VGCCs are tightly coupled to the Ca^2+^ sensor proteins associated with exocytosis at 10 to 20 nanometers in GABAergic synapses [80] (like the stellate cell synapses in Figure 3). Additionally, in postnatal day 21 rats, the calyx of Held synapses are insensitive to EGTA [78]. In contrast to these synapses, synapses between hippocampal mossy fiber terminals and CA3 pyramidal cells are sensitive to BAPTA and EGTA [89]. VGCCs and Ca^2+^ sensor proteins are estimated to be located approximately 80 nanometers apart and loosely coupled at these synapses [89] (like the granule cell synapses in Figure 3). On the other hand, NMJs are not responsive to BAPTA or EGTA, i.e., neurotransmitter release is maintained in the presence of BAPTA and EGTA [90,91]. According to these results, VGCCs and Ca^2+^ sensor proteins are very tightly coupled at NMJs. Thus, the smallest distance between VGCCs and Ca^2+^ sensor proteins is observed at NMJs, a medium distance is observed at the calyx of Held or GABAergic inhibitory synapses, and the largest distance is observed at hippocampal mossy fiber synapses. The opening of VGCCs forms a transient Ca^2+^ concentration distribution inside of the presynaptic membrane near the VGCCs (<100 nm), referred to as “Ca^2+^ nanodomains”, and away from the VGCCs (>100 nm), referred to as “Ca^2+^ microdomains” [92]. In summary, Ca^2+^ sensor proteins are present in Ca^2+^ nanodomains or in Ca^2+^ microdomains, depending on the type of central synapse, while Ca^2+^ sensor proteins in NMJs are located immediately adjacent to the VGCCs.

How is the distance between VGCCs and Ca^2+^ sensor proteins regulated? Active zone proteins are assumed to play a key role in this process because active zone proteins are scaffolding proteins that bind directly or indirectly to VGCCs, synaptic vesicle proteins, and SNARE proteins to form presynaptic molecular complexes (Figure 1). Therefore, determining the distance between VGCCs and active zone proteins is essential for understanding the mechanisms that regulate exocytosis. However, confirmation of the distances between multiple presynaptic proteins simultaneously is technically challenging because the estimated distances are very small (tens of nanometers). A breakthrough in measuring the spatial distribution of presynaptic proteins was achieved using sodium dodecyl sulfate-digested freeze-fracture replica labeling (SDS-FRL), a highly sensitive and quantitative method used to determine the two-dimensional arrangement of membrane molecules on the scale of 10 nanometers [93]. Simultaneous spatial analysis of P/Q-type VGCCs and RIM1 and RIM2, which directly bind to synaptic vesicles containing Ca^2+^ sensor proteins via Rab3s [31], with SDS-FRL easily confirmed that these proteins are only localized in the active zone and in close proximity, i.e., within a few tens of nanometers, at glutamatergic synapses in the CA3 region of the rat hippocampus [4]. At parallel fiber synapses in the cerebellum, P/Q-type VGCCs are located within a few tens of nanometers of active zone proteins [79], such as RIM and ELKS, which bind to synaptic vesicles containing Ca^2+^ sensor proteins via Rab6s [28,94], and the presynaptic cell adhesion protein neurexin [79]. Therefore, these results indicate that VGCCs and active zone proteins are located in close proximity within a few tens of nanometers, supporting that VGCCs and Ca^2+^ sensor proteins may be in close proximity in the order of tens of nanometers via active zone proteins.

Furthermore, Munc13-1 labeling was used to identify a putative synaptic vesicle release site, and SDS-FRL measurements revealed that the distance between P/Q-type VGCCs and Munc13-1 determines differences in the release probability of neurotransmitters at excitatory and inhibitory synapses in the cerebellum [81]. The weak synapses were excitatory synapses with a low release probability (approximately 0.2) formed between granule and Purkinje cells [81,95,96]. The strong synapses were inhibitory synapses with a high release probability (0.3–0.8) formed between stellate cells [61,81,97]. Munc13-1 clusters were tightly coupled to a cluster of P/Q-type VGCCs at strong synapses (approximately 10 nanometers), whereas Munc13-1 clusters were not in the vicinity of P/Q-type VGCCs, and P/Q-type VGCCs did not form clusters at weak synapses (approximately 50 nanometers) [81,98] (Figure 3). Thus, the diversity of release probabilities at excitatory synapses formed between granule and Purkinje cells and inhibitory synapses formed between stellate cells is based on the spatial arrangement of P/Q-type VGCCs and Munc13-1 within one synapse.

Unexpectedly, the number of VGCCs was three times higher at weak synapses than at strong synapses [81]. Additionally, action potential-induced Ca^2+^ transients were four times larger in weak synaptic boutons than in strong synaptic boutons [81]. These results indicate that the difference in release probability between strong and weak synapses cannot be explained by the number of VGCCs and the amount of Ca^2+^ influx. Therefore, the measurement of the distance between synaptic proteins is an indispensable approach to reveal the molecular mechanisms underlying neurotransmitter release properties.

**Figure 4:**
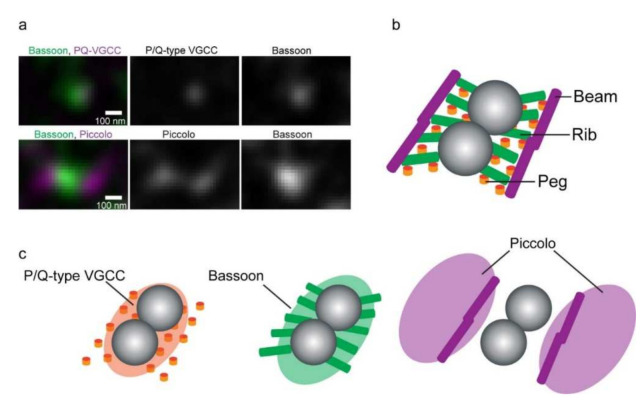
Active zone proteins and structure of mouse NMJs. (**a**) Dual-color STED micrographs show an overlap of Bassoon (green) and P/Q-type VGCCs (magenta) and the side-by-side distribution pattern of Bassoon (green) and Piccolo (magenta). (**b**) The structural model of a mouse NMJ active zone was elucidated using electron microscopy (EM) tomography [100]. (**c**) A theoretical overlay of the active zone protein distribution pattern on the active zone structure model. The “pegs” in the EM tomography model are considered transmembrane channel proteins, which may include P/Q-type VGCCs. The side-by-side distribution pattern of Piccolo and Bassoon resembles the distribution pattern of structures named the “beams” and the “ribs” in the EM tomography model. These figures were adopted from Nishimune et al., Sci. Rep. 2016, 6, 27935 [5], and the EM tomography model was adopted with modification from Nagwaney et al., J. Comp. Neurol., 2009, 513, 457 [100] (with permission from John Wiley & Sons, Inc., license number: 5191011487547).

At NMJs, an SDS-FRL analysis of the spatial distribution of VGCCs and active zone proteins was not performed. Instead, we performed dual-color imaging of VGCCs, Bassoon, and Piccolo using stimulated emission depletion (STED) super-resolution microscopy [3], which has been used to analyze the distribution of molecules at 30–50 nanometer resolution. This study represents the first spatial analysis of these molecules at mammalian NMJs with super-resolution microscopy [5]. The distribution patterns of Bassoon and P/Q-type VGCCs were punctate and discrete, and the two proteins were colocalized (Figure 4a). This result is consistent with the results of an immunoprecipitation study showing that Bassoon directly interacts with the β1b- and β4-subunits of VGCCs [34], and the finding that Bassoon localizes with P/Q-type VGCCs in the active zone via RIM-binding proteins in the hippocampus [99]. In addition, we found that Piccolo puncta sandwiched a Bassoon punctum in a side-by-side pattern [5] (Figure 4a). This side-by-side pattern is similar to the active zone model based on electron tomography data from an analysis of the macromolecules comprising the active zone of mouse NMJs [100]. The electron tomography data showed that the macromolecules that compose active zone material called “pegs”, “ribs”, and “beams” are associated with two synaptic vesicles [100] (Figure 4b). This structural pattern is similar to the distribution pattern of the P/Q-type VGCCs, Bassoon, and Piccolo identified using STED microscopy [5] (Figure 4c). Some of the pegs, ribs, and beams may contain P/Q-type VGCCs, Bassoon, and Piccolo, suggesting that a single P/Q-type VGCC cluster localizes at a docking site for two synaptic vesicles at active zones of mouse NMJs. In another study, light microscopy revealed that the average number of Bassoon puncta at mouse NMJs was 850, and electrophysiological studies estimated that the average RRP size was 1730 [15]. These results support the hypothesis that a single active zone forms a functional docking site for two synaptic vesicles at mouse NMJs.

Interestingly, the quantal content detected in a single action potential at NMJs was much lower than that estimated by the RRP [15]. Synaptic vesicle release from only a subset of active zones within one NMJ has also been directly observed using fluorescence probes [101,102,103,104]. Thus, the active zones within one NMJ have different release characteristics. The mechanism underlying the functional heterogeneity of active zones within one synapse has not yet been elucidated. Nevertheless, subtle differences in the spatial arrangement of P/Q-type VGCCs and active zone proteins in each active zone, heterogeneity in the open probability of P/Q-type VGCCs, or other proteins required for exocytosis that affect the release probability may exist, which may lead to diversity in the characteristics of neurotransmitter release at NMJs.

### 2.5. Nanocolumn Structures Linking Pre- and Postsynaptic Sites

The spatial arrangement of pre- and postsynaptic connections is also critical for efficient synaptic transmission. Recently, a super-resolution microscopy analysis revealed that active zone proteins at central synapses and NMJs aligned precisely with postsynaptic receptors at the nanoscale level [6,105].

Spatial analysis of RIMs using 3D stochastic optical reconstruction microscopy (STORM) showed that RIMs formed clusters with a diameter of approximately 80 nanometers near active zones at the glutamatergic presynaptic terminal in cultured rat hippocampal neurons [6]. The RIM clusters were located within 40 nanometers of the synaptic vesicle fusion site, which was visualized using the fluorescent probe vGlut1-pHluorin-mCherry [106,107], indicating the fusion of vesicles with the membrane [6]. Furthermore, each cluster of RIMs and the postsynaptic scaffolding protein PSD-95 were spatially aligned [6]. The researchers hypothesized that the formation of a “transsynaptic nanocolumn” structure facilitates the efficient release of neurotransmitters in the vicinity of postsynaptic receptors.

Similar nanocolumn structures have been identified in cultured neurons and brain slices from the rodent cerebral cortex, i.e., presynaptic vesicular glutamate transporter 1 (vGlut1) clusters containing synaptophysin 1 or Bassoon aligned with postsynaptic PSD-95 clusters [108]. Surprisingly, the average size of individual vGlut1, synaptophysin 1, Bassoon, and PSD-95 clusters did not vary between single and multi-cluster spines [108]. Furthermore, chemically induced long-term potentiation increased the spine size, the mobility of clusters that remained aligned between pre- and postsynaptic sites, and the number of pre- and postsynaptic clusters [108]. Thus, changes in the number of nanocolumns might be a molecular mechanism underlying synaptic plasticity and heterogeneity in the transmission efficiency of individual synapses.

Then, which molecules contribute to the formation of nanocolumns? Neuroligins at the postsynaptic site were identified as candidates and the focus of one study [109]. Neuroligins are known to connect pre- and postsynaptic terminals via a presynaptic binding partner protein, neurexin. The expression of a C-terminally truncated neuroligin 1 mutant unable to bind to PSD-95 resulted in mild separation of the presynaptic RIM cluster and the postsynaptic AMPA receptor cluster in cultured neurons and CA1 pyramidal neurons of slice preparations from the rodent hippocampus [109]. The amplitude of AMPA receptor-mediated miniature EPSCs and evoked EPSCs was reduced accordingly [109]. Thus, transsynaptic nanocolumns probably form via neuroligin-neurexin complexes to tightly regulate synaptic efficacy.

Similarly, in inhibitory synapses in the rodent hippocampus and spinal cord, transsynaptic nanocolumns in which presynaptic RIM clusters were aligned with postsynaptic gephyrin and GABA A receptor clusters were observed [110,111]. Therefore, inhibitory synapses also form transsynaptic nanocolumns in which clusters of active zone proteins are precisely aligned with postsynaptic receptor clusters.

NMJs are structurally unique, as the postsynaptic muscle membrane forms junctional folds in the sarcolemma, effectively increasing the surface area of the membrane with acetylcholine receptors clustered on the crests of the folds. Spatial analysis using 3D structured illumination microscopy (SIM) and 3D STORM revealed that postsynaptic acetylcholine receptors are not uniformly distributed across the crest of junctional folds, but instead, locally enriched at the edges, aligning with the presynaptic Piccolo puncta [105]. Furthermore, electron microscopy analysis revealed that 78% of the presynaptic active zones align with junctional folds [112]. This nanoscale organization of active zones and acetylcholine receptor clusters may form transsynaptic nanocolumns for effective synaptic transmission.

## 3. Roles of Exocytosis-Related Proteins during Aging and in Neurological Diseases

Synaptic transmission is essential for the maintenance of high-order brain functions such as cognition, learning, and memory, and for the control of motor functions such as walking and breathing. Therefore, disruption of the neurotransmitter release function has been implicated in various neurological diseases. The molecular mechanism underlying the disruption of neurotransmitter release in individuals with neurological disorders was shown with toxins [113,114] and autoantibodies [115,116] targeting exocytosis-related presynaptic proteins. Furthermore, alterations in genes encoding active-zone proteins, synaptic vesicle proteins, and SNARE proteins may interfere with neurotransmitter release and cause symptoms of peripheral and central nervous system diseases [9,10,11,12]. Recently, aging was revealed to cause not only a decrease in the number of synapses and synaptic connections, but also a decrease in the expression of presynaptic proteins critical for synaptic transmission, leading to decreased muscle strength and cognitive function. In this section, we describe the relationship between presynaptic molecules and the symptoms of peripheral and central nervous system diseases, and the similarities and differences in the molecular mechanisms that induce these symptoms.

### 3.1. Molecules Inhibiting Exocytosis-Related Proteins and Neurological Diseases of the Peripheral and Central Nervous Systems

Clostridial neurotoxins, such as botulinum toxin (BoNT) and tetanus toxin (TeNT), have long been known to dysregulate muscle contractile function [117,118,119]. However, the clinical symptoms of botulism and tetanus are very different. Botulism causes flaccid paralysis, in which muscles are unable to contract. Tetanus, on the other hand, causes spastic paralysis characterized by periodic hypercontractions of skeletal muscles. In 1992, the molecular mechanism underlying the clinical symptoms induced by clostridial neurotoxins was discovered to be mediated by the cleavage of SNARE proteins [113,114]. The discovery of these neurotoxic effects supports the hypothesis that SNARE proteins are essential for exocytosis and that clostridial neurotoxins cause synaptic diseases by impairing exocytotic function.

Differences in clinical symptoms are based on differences in the destinations of BoNTs and TeNT after they enter the nerve terminals. BoNTs and TeNT enter nerve terminals through endocytosis by binding to gangliosides, lipids, and synaptic vesicle proteins such as synaptotagmin and synaptic vesicle glycoprotein 2 [119,120,121]. After entering the nerve terminals, BoNTs are taken up by endosomes. At this time, the N-terminal domain of the heavy chain is presumed to form a pore in the endosomal membrane, and the disulfide bonds of the BoNT exposed in the cytoplasm are reduced, causing the light chain to dissociate from the heavy chain and be released into the cytoplasm [122,123,124,125,126]. The light chains specifically cleave a group of SNARE proteins in motor nerve terminals. BoNTs are classified into serotypes A through G, which target different SNARE proteins. BoNT type A and BoNT type E cleave SNAP-25; BoNT type B, BoNT type D, BoNT type F, and BoNT type G cleave synaptobrevin; and BoNT type C cleaves SNAP-25 and syntaxin 1 [119]. The cleavage of these SNARE proteins inhibits the release of neurotransmitters in NMJs, leading to flaccid paralysis.

TeNT, on the other hand, is taken up by the endosomes of motor nerve terminals and then transported retrogradely along the axons to motor neuron cell bodies in the spinal cord, where it is transsynaptically incorporated into the presynaptic terminals of upstream neurons [121]. These upstream neurons form inhibitory central synapses that regulate motor neuron activity [119]. At inhibitory presynaptic terminals, TeNT cleaves the SNARE protein synaptobrevin [113], thereby blocking inhibitory neurotransmission within the spinal cord. As a result, motor neurons become desuppressed, leading to muscle spasms and spastic paralysis. The difference between BoNTs activated at NMJs and TeNT activated at inhibitory synapses in the spinal cord reflects the difference in clinical symptoms between flaccid and spastic paraplegia. The common feature of these toxins is that they rarely impair brain functions, because neither toxin crosses the blood–brain barrier [127] and TeNT only reaches inhibitory synapses in the spinal cord or brainstem via transsynaptic incorporation.

Neurological diseases caused by molecules targeting exocytosis-related proteins may be induced not only by exogenous toxins, but also by endogenous autoantibodies. One such autoimmune disease is Lambert–Eaton myasthenic syndrome (LEMS); autoantibodies targeting P/Q-type VGCCs are produced in approximately 90% of patients with LEMS [116]. LEMS is an autoimmune disease characterized by muscle weakness in proximal limb muscles and autonomic dysfunction. Electrophysiological and pharmacological studies in mice have shown that autoantibodies against P/Q-type VGCCs reduce neurotransmitter release function at NMJs [128,129]. This disruption of presynaptic function is explained by the fact that the major VGCCs in motor nerve terminals in rodents and humans are P/Q-type VGCCs [75,76]. As a result, acetylcholine release from nerve terminals is impaired, resulting in symptoms such as muscle weakness [128] and autonomic neuropathy [130]. The mechanism underlying the disruption of acetylcholine release in motor nerve terminals may be based on a decrease in the number of P/Q-type VGCCs. One piece of evidence supporting this hypothesis is that the number of P/Q-type VGCCs is selectively reduced in the cerebellar molecular layer in patients with LEMS presenting cerebellar ataxia on autopsy [131].

### 3.2. Genetic Alterations in Exocytosis-Related Proteins and Neurological Diseases

Human exome analysis, genome-wide association studies, and copy number variation analysis have identified disease susceptibility genes in patients with neurological diseases such as neurodevelopmental disorders, neuromuscular disorders, and neuropsychiatric disorders [132,133,134]. As a result, genetic alterations in various exocytosis-related proteins were identified in these patients [9,10,11,12,135,136,137,138,139,140,141,142,143,144,145,146]. There is accumulating evidence for the relationship between these mutations and disease (Table 3), and the effects of several of these mutations on neurotransmitter release have been determined using genetically modified mice and neurons.

Genetic mutations in members of different protein families have been identified in multiple diseases. Synaptobrevin protein family members, i.e., synaptobrevin 1 and synaptobrevin 2, and synaptotagmin protein family members, i.e., synaptotagmin 1 and synaptotagmin 2, share a high degree of sequence homology among the family members and have a common ability to induce exocytosis. Interestingly, genetic mutations in synaptobrevin 1 [12,135,136] and synaptotagmin 2 [10,142,143,144,145] result in similar symptoms. In humans, dysfunction of synaptobrevin 1 causes movement disorders such as motor retardation, muscle weakness, and spastic ataxia with a presynaptic impairment at NMJs [12,135,136], while mutations in synaptotagmin 2 also result in motor disorders with a deficit in acetylcholine release at NMJs [10,142,143,144,145]. Furthermore, genetic mutations in synaptobrevin 2 [137,138] and synaptotagmin 1 [139,140,141] result in a different set of similar symptoms. Dysfunction of synaptobrevin 2 causes neurodevelopmental disorders such as autistic features, developmental delay, and moderate to severe intellectual disability [137,138], while mutations in synaptotagmin 1 result in neurodevelopmental disorders such as profound cognitive impairment, lack of eye contact, and severe motor delay [139,140,141].

The mechanisms by which genetic mutations in different members of the same protein family cause different symptoms may be based on differences in the expression patterns and temporal functions of each member in tissues. In the rodent central nervous system, synaptobrevin 1 is expressed at the highest levels in the cerebellum, midbrain, diencephalon, and spinal cord; however, its expression is limited in the cerebrum [147,148,149,150,151]. In the hippocampus, synaptobrevin 1 is preferentially expressed at inhibitory synapses [152,153]. In the peripheral nervous system, synaptobrevin 1 is expressed at high levels in cranial nerves and NMJs [149,150,151,154]. On the other hand, synaptobrevin 2 is a major v-SNARE protein in the central nervous system and is detected at the highest levels in the frontal lobe and putamen in the postmortem human brain [137]. Studies in rodents suggest that synaptobrevin 2 is expressed at high levels in autonomic and sensory nerves in the peripheral nervous system and at low levels at NMJs [149]. Synaptotagmin 1 is most abundant at central synapses in the cerebral cortex, hippocampus, striatum, and midbrain, and at synapses in sensory and autonomic nerves [155,156,157]. Synaptotagmin 2 is preferentially expressed at NMJs and in the spinal cord, brainstem, and cerebellum [156,158]. It has also been shown to be expressed at inhibitory synapses in the cerebellum [159]. In addition, the temporal pattern of functions of members of the same protein family may differ, as indicated by phenotypic differences in mice with knockout of each gene. *Synaptobrevin 1* knockout mice appear to be generally normal during the first week of life [148,150]. By the second week of life, they show marked motor deficits and die within three weeks of birth [148,150], while *Synaptobrevin 2* knockout mice die shortly after birth [160]. *Synaptotagmin 1* knockout mice die one to two days after birth [85], while *Synaptotagmin 2* knockout mice appear to be generally normal during the first week of life, but show marked motor deficits by the second week of life and die within three weeks of birth [158]. Due to differences in the expression patterns of these proteins and the timing of their functions, mutations in synaptobrevin 2 and synaptotagmin 1 probably tend to cause neurodevelopmental disorders, while mutations in synaptobrevin 1 and synaptotagmin 2 tend to cause motor disorders.

As we discussed in the preceding sections, Munc13-1 is widely expressed at NMJs and central synapses and plays an important role in exocytosis [32,42,43]. A homozygous nonsense mutation in *Munc13-1* was identified in a patient suffering from a fatal syndrome of microcephaly, cortical hyperexcitability, and myasthenia [146]. This mutation results in the truncation of Munc13-1 at amino acid residue 101 [146]. The marked perturbation of cortical and neuromuscular synaptic transmission might be because Munc13-1 is widely expressed at central synapses and NMJs [32,42,43]. However, the cortical hyperexcitability of this patient at four months of age was inconsistent with the marked reduction in central excitatory synaptic transmission in Munc13-1-deficient mice [42]. One of the mechanisms underlying this cortical hyperexcitability may have been abnormalities in the “GABA switch” [161,162,163], which is a change in the polarity of GABA signaling from excitatory to inhibitory within the first few weeks of life [161,162,163]. The patient with the nonsense mutation in *Munc13-1* may have had abnormalities in the GABA switch, leading to a shift in the balance between excitation and inhibition of neural activity toward hyperexcitability, as the patient was born prematurely at 32 weeks of gestation [146].

A de novo heterozygous missense mutation (P814L) in Munc13-1 was also detected in a patient with delayed neurological development, dyskinesia, autism spectrum disorder, and comorbid attention-deficit hyperactivity disorder [11]. Electrophysiological studies of mouse neurons have revealed that this mutation in *Munc13-1* is a gain-of-function mutation that causes an increase in the fusion propensity of synaptic vesicles [11]. This increase in fusion propensity leads to an increased synaptic vesicle release probability and abnormal short-term synaptic plasticity [11]. Thus, this gain-of-function mutation in *Munc13-1* causes neurodevelopmental disorders with different clinical manifestations from those caused by the loss-of-function mutation [146] described in the previous paragraph.

ELKS is associated with autism spectrum disorders and childhood apraxia of speech. Patients with these disorders exhibit microdeletions of chromosome 12p13.33. A comparison of these microdeletions identified common deletions in the *ELKS* gene [9]. Interestingly, all patients with these microdeletions showed delayed neurodevelopment. Some children also showed deficits in gross motor, fine motor, and oral motor function [9]. These neurodevelopmental delays and motor dysfunction likely resulted from a lack of ELKS in the brain and NMJs [63,64]. Therefore, ELKS deletion may impair the regulatory function of muscles involved in vocalization and movements.

The causative relationship between neurological symptoms and gene mutations or reduced functions of active zone proteins, synaptic vesicle proteins, and SNARE proteins may be better understood by analyzing the preferential localization and temporal functions of these proteins in the synapses of the peripheral and/or central nervous systems. Therefore, these proteins are promising targets for therapeutic interventions for physiological aging or neurological diseases for which efficacious therapies are currently lacking.

### 3.3. Presynaptic Structure and Aging

Physiological aging is a major risk factor for neuromuscular and neurological diseases characterized by sarcopenia and mild cognitive decline. One of the causes of this age-related decline in biological function is the impairment of synaptic transmission [164,165]. We performed dual-color super-resolution imaging of P/Q-type VGCCs, Bassoon, and Piccolo in mouse NMJs and found one of the characteristic changes in molecular spatial distribution during aging [5]. P/Q-type VGCCs and Bassoon colocalized in the NMJs of adult mice, and Piccolo puncta sandwiched Bassoon puncta [5]. However, the protein levels of P/Q-type VGCCs and Bassoon were significantly decreased in the NMJs of aged mice [5]. Interestingly, the expression level of Piccolo was approximately the same in adult mice and aged mice [5]. A selective reduction in the levels of active zone proteins may be one possible cause of impaired synaptic transmission at NMJs and sarcopenia in aged mice. The mechanisms of NMJ impairment upon aging are summarized in our previous review article [166].

Impaired synaptic transmission at central synapses during physiological aging is likely to affect cognitive functions such as learning and memory. A decrease in the number of synapses in the cerebral cortex and hippocampus has been suggested to underlie the age-related decline in cognitive functions. Age-related changes in the number of excitatory (asymmetric) and inhibitory (symmetric) synapses were reported in layers 2/3 and 5 of area 46 in the prefrontal cortices of rhesus monkeys using quantitative electron microscopy [164]. Approximately 30% of all types of synapses in layers 2/3 were lost between the ages of 5 and 30 years, and both excitatory and inhibitory synapses were lost at the same rate [164]. The reduction in the number of layer 2/3 synapses correlated with performance on memory tests and the cognitive impairment index [164]. On the other hand, the number of synapses in layer 5 was reduced by 20%, mostly due to a reduction in the number of excitatory synapses [164]. However, no correlation between the decrease in the number of excitatory synapses in layer 5 and cognitive decline was identified [164]. In the dentate gyrus and CA3 region of the rat hippocampus, the number of synapses also markedly decreases with age [167,168]. In contrast, the synaptic density in the CA1 region does not change with age [169]. Therefore, the age-related decrease in synapse number is site-specific.

Synaptic bouton types include single-synapse boutons, multiple-synapse boutons, and nonsynaptic boutons. These types of synaptic boutons in the outer molecular layer of the dentate gyrus in the hippocampi of rhesus monkeys change with age, as revealed by electron microscopy [170]. The percentage of nonsynaptic boutons in aged monkeys (mean, 29.13 years old) was twice that in young adult monkeys (mean, 9.72 years old) [170]. This index correlated with age-related memory impairment [170]. In contrast, aged monkeys had fewer multiple-synapse boutons and synaptic connections per multiple-synapse bouton than young adult monkeys, and this index correlated inversely with age-related memory impairment [170]. Based on these results, age-related changes in synaptic bouton types in the outer molecular layer may be associated with memory decline. An ultrastructural analysis of mossy fiber-CA3 synapses revealed that the area occupied by mossy fiber boutons remained unchanged, while the density of synaptic vesicles was reduced by approximately 30% in aged mice compared with young mice [171]. Similarly, aging caused a decrease in the density of synaptic vesicles at CA3–CA1 synapses [171]. Moreover, a significant decrease was observed in the density of distal synaptic vesicles (150 nanometers away from the active zone) in the hippocampal synapses of 24- to 27-month-old rats compared with those of 3- to 5-month-old rats [172].

Changes in the profiles of synaptic proteins during aging were determined using proteomic analysis of synaptosomes in rodents and humans. A total of 273 proteins showed age-related changes in expression in synaptosomes from the hippocampi of young (three months), adult (12 months), and aged (26 months) rats [173]. The pathway analysis of these proteins revealed that the most highly regulated network was neurotransmission-regulating proteins [173]. The reduction in synapsin 1 expression between aged rats (26 months) and young rats (three months) identified by the proteomic analysis was confirmed by performing immunoblotting of soluble proteins extracted from synaptosome samples [173]. Synapsins are responsible for maintaining and stabilizing synaptic vesicles in a reserve pool distant from the active zone [174]. The density of distal synaptic vesicles was reduced in *synapsin 1* knockout mice [175,176,177]. The phenotype of *synapsin 1* knockout mice was consistent with the marked decrease in the density of distal synaptic vesicles in the hippocampal synapses of aged rats [172]. In addition, the expression levels of the SNARE proteins synaptobrevin 2, syntaxin 1, and SNAP-25 were similarly decreased by 15% to 35% with increasing age compared to the levels in adult rats [173]. In addition, a proteomic analysis of the postmortem dorsolateral prefrontal cortices of aged humans showed decreased expression of proteins such as syntaxin 1A, synaptotagmin 1, Bassoon, and vGlut1 in elderly individuals with fast cognitive decline compared to elderly individuals with stable cognitive function throughout life [13]. The decrease in the expression of these synaptic proteins may have been responsible for age-related impairment in synaptic transmission. In rodents, a reduction in the expression of SNARE proteins and synapsins was reported to cause epilepsy [174,175,178,179,180,181], consistent with the frequent occurrence of epilepsy in elderly people.

However, systems that compensate for the decline in synaptic transmission are also present during physiological aging. For example, in aged rats, an increase in the quantal size of individual synapses has been found to compensate for a decrease in the number of synapses in the hippocampal perforant path [182]. In aged monkeys, an increase in the number of synaptic vesicles at inhibitory axosomatic synapses was observed to compensate for the decrease in the number of synapses in the prefrontal cortex [183]. Thus, clarification of the changes in the number of synapses and presynaptic proteins at excitatory and inhibitory synapses in future studies is expected to provide new insights into the mechanism underlying the decline in motor function and cognition related to physiological aging.

## Figures and Tables

**Table 1:** Structural features of active zones at the presynaptic terminal.

Synapse	Species	Age	Active Zone Number Per Presynaptic Terminal	Presynaptic Terminal Size	Active Zone Density	Active Zone Size	Reference
NMJ	Mouse	Day 54	780	295 μm^2^	2.6/μm^2^	0.082 μm^2^	[17]
NMJ	Mouse	Adult	850	335.9 μm^2^	2.5/μm^2^ *	N.P.	[15]
NMJ	Human	67 years	N.P.	122.7 μm^2^	N.P.	N.P.	[18]
NMJ	Human	Adult	N.P.	N.P.	2.6/μm^2^	N.P.	[19]
Stratum radiatum in CA1 hippocampus	Mouse	Adult	one for more than 90% of synapses	0.086 μm^3^	13.4/μm^3^ *	0.039 μm^2^	[20]
Piriform cortex layer 1a	Mouse	7 months	one for more than 90% of synapses	0.367 μm^3^	3.1/μm^3^ *	0.095 μm^2^	[21]
Piriform cortex layer 1b	Mouse	7 months	one for more than 90% of synapses	0.208 μm^3^	5.5/μm^3^ *	0.097 μm^2^	[21]
Calyx of Held	Rats	Day 9	554	1022 μm^2^	0.54/μm^2^ *	N.P.	[22]
Calyx of Held	Rats	Day 14	678	N.P.	N.P.	0.089 μm^2^	[24]

* Calculated from data in the papers. N.P.: not provided.

**Table 2:** Functional changes in NMJs and central synapses upon knockout of active zone proteins.

Synapse	Gene Knockout	Excitatory/Inhibitory	Evoked EPSC/IPSC/EPP Amplitude	Miniature EPSC/IPSC/EPP Amplitude	Miniature EPSC/IPSC/EPP Frequency	Release Probability	RRP Size	Reference
Schaffer collateral to hippocampal CA1 pyramidal cell synapses (3- to 7-week) and cultured hippocampal synapses	*Rim1a* and *Rim1b*	Excitatory	N.P.	No change	Decrease	Decrease	N.P.	[37,38]
Hippocampal CA1 synapses (3- to 7-week) and cultured hippocampal synapses	*Rim1a* and *Rim1b*	Inhibitory	Decrease	No change	Decrease	Decrease	Decrease	[37,38]
Calyx of Held synapses in brainstem slices (Postnatal day 9–11)	*Rim1* and *Rim2*	Excitatory	1/5-fold decrease	No change	N.P.	~25% decrease	75% decrease	[39]
Cultured hippocampal synapses	*Rim1* and *Rim2*	Excitatory	1/10-fold decrease	No change	1/10-fold decrease	N.P.	1/3- to 1/4-fold decrease	[40,41]
Cultured hippocampal synapses	*Rim1* and *Rim2*	Inhibitory	1/10-fold decrease	No change	1/3-fold decrease	Decrease	1/3- to 1/4-fold decrease	[40,41]
Diaphragm NMJs (Embryonic day 18.5)	*Rim1a* and *Rim2a*	Excitatory	1/10-fold decrease	No change	No change (decrease in 40 mM KCl)	Decrease	N.P.	[33]
								
Cultured hippocampal synapses	*Munc13-1*	Excitatory	Markedly decrease	No change	Decrease	No change	Markedly decrease	[42]
Cultured hippocampal synapses	*Munc13-1*	Inhibitory	No change	N.P.	N.P.	N.P.	No change	[42,43]
Cultured hippocampal synapses	*Munc13-2*	Excitatory	No change	No change	No change	N.P.	No change	[43]
Cultured hippocampal synapses	*Munc13-2*	Inhibitory	No change	No change	No change	N.P.	N.P.	[43]
Cultured hippocampal synapses	*Munc13-1* and *Munc13-2*	Excitatory	Completely abolish	Completely abolish	Completely abolish	N.P.	Completely abolish	[43]
Cultured hippocampal synapses	*Munc13-1* and *Munc13-2*	Inhibitory	Completely abolish	Completely abolish	Completely abolish	N.P.	N.P.	[43]
Diaphragm NMJs (Embryonic day 18.5)	*Munc13-1* and *Munc13-2*	Excitatory	1/16-fold decrease	No change	More than 2-fold increase	Decrease	Decrease	[32]
								
Schaffer collateral to hippocampal CA1 pyramidal cell synapses (4- to 6-week) and cultured hippocampal synapses	*ELKS2a*	Excitatory	N.P.	No change	No change	No change	No change	[44]
Hippocampal CA1 synapses (4- to 6-week) and cultured hippocampal synapses	*ELKS2a*	Inhibitory	Increase	No change	No change	Increase	Increase	[44]
Calyx of Held synapses in brainstem slices (Postnatal day 16–21)	*ELKS1* and *ELKS2*	Excitatory	No change	No change	Increase	Increase	Decrease	[45]
Cultured hippocampal synapses	*ELKS1a* and *ELKS2a*	Excitatory	1/2-fold decrease	No change	1/2-fold decrease	No change	Decrease	[46]
Cultured hippocampal synapses	*ELKS1a* and *ELKS2a*	Inhibitory	1/2-fold decrease	No change	1/2-fold decrease	Decrease	No change	[46,47]

N.P.: not provided.

**Table 3:** Relationship between genetic alterations in exocytosis-related proteins and neurological disease symptoms.

Gene	Mutation	Disease Symptoms	Reference
Synaptobrevin 1	G18Wfs*5 R49P chr12:g. 6,574,054 T > G; disruption of mRNA splicing and generation of S114R variant in synaptobrevin 1 isoform D	Congenital myasthenic syndrome, motor retardation, muscle weakness, spastic ataxia, gait disturbance	[12,135,136]
Synaptobrevin 2	V43del I45del A67P S75P P77S E78A	Autistic features, developmental delay, moderate to severe intellectual disability, poor visual fixation, absent purposeful hand movements	[137,138]
Synaptotagmin 1	M303K D304G D366E I368T N371K	Profound cognitive impairment, lack of eye contact, severe motor delay, developmental delay varying in severity from moderate to profound	[139,140,141]
Synaptotagmin 2	Exon3-9del V243Gfs*13 E269 * D307A P308L Y309 * I371K R397Sfs*37	Distal hereditary motor neuropathy, congenital myasthenic syndrome, foot deformity since childhood, distal limb weakness, areflexia, gait abnormality	[10,142,143,144,145]
Munc13-1	Q102 *	Microcephaly, cortical hyperexcitability, fatal myasthenia	[146]
	P814L	Delayed neurological development, dyskinesia, autism spectrum disorder, comorbid attention-deficit hyperactivity disorder	[11]
ELKS	Microdeletions of chromosome 12p13.33 including ELKS gene	Autism spectrum disorder, childhood apraxia of speech, deficits in gross motor, fine motor, and oral motor function	[9]

fs: frameshift, *: stop codon, del: deletion.

## Data Availability

Not applicable.
